# Selenium, but Not Lycopene or Vitamin E, Decreases Growth of Transplantable Dunning R3327-H Rat Prostate Tumors

**DOI:** 10.1371/journal.pone.0010423

**Published:** 2010-04-29

**Authors:** Brian L. Lindshield, Nikki A. Ford, Kirstie Canene-Adams, Alan M. Diamond, Matthew A. Wallig, John W. Erdman

**Affiliations:** 1 Division of Nutritional Sciences, University of Illinois at Urbana-Champaign, Urbana, Illinois, United States of America; 2 Department of Food Science and Human Nutrition, University of Illinois at Urbana-Champaign, Urbana, Illinois, United States of America; 3 Department of Pathobiology, University of Illinois at Urbana-Champaign, Urbana, Illinois, United States of America; 4 Department of Pathology, College of Medicine, University of Illinois at Chicago, Chicago, Illinois, United States of America; New Mexico State University, United States of America

## Abstract

**Background:**

Lycopene, selenium, and vitamin E are three micronutrients commonly consumed and supplemented by men diagnosed with prostate cancer. However, it is not clear whether consumption of these compounds, alone or in combination, results in improved outcomes.

**Methodology/Principal Findings:**

We evaluated the effects of dietary lycopene (250 mg/kg diet), selenium (methylselenocysteine, 1 mg/kg diet), and vitamin E (γ-tocopherol, 200 mg/kg diet) alone and in combination on the growth of androgen-dependent Dunning R3327-H rat prostate adenocarcinomas in male, Copenhagen rats. AIN-93G diets containing these micronutrients were prefed for 4 to 6 weeks prior to tumor implantation by subcutaneous injection. Tumors were allowed to grow for ∼18 weeks. Across diet groups, methylselenocysteine consumption decreased final tumor area (*P* = 0.003), tumor weight (*P* = 0.003), and the tumor weight/body weight ratio (*P* = 0.003), but lycopene and γ-tocopherol consumption intake did not alter any of these measures. There were no significant interactions among nutrient combinations on tumor growth. Methylselenocysteine consumption also led to small, but significant decreases in body weight (*P* = 0.007), food intake (*P* = 0.012), and body weight gain/food intake ratio (*P* = 0.022). However, neither body weight nor gain/food intake ratio was correlated with tumor weight. Methylselenocysteine, lycopene, and γ-tocopherol consumed alone and in combination did not alter serum testosterone or dihydrotestosterone concentrations; tumor proliferation or apoptosis rates. In addition, the diets also did not alter tumor or prostate androgen receptor, probasin, selenoprotein 15, selenoprotein P, or selenium binding protein 2 mRNA expression. However, using castration and finasteride-treated tissues from a previous study, we found that androgen ablation altered expression of these selenium-associated proteins.

**Conclusions:**

Of the three micronutrients tested, only methylselenocysteine consumption reduced growth of transplantable Dunning R3327-H prostate tumors, albeit through an unresolved mechanism.

## Introduction

Prostate cancer is the most frequently diagnosed male cancer in the United States, accounting for about one quarter of all male cases in 2009 [1]. Selenium, vitamin E, and lycopene, the red-pigmented carotenoid found primarily in tomatoes, are among the most commonly used supplements by men diagnosed with prostate cancer [2], [3]. Despite their use by prostate cancer patients, it has not been established whether supplementation of these micronutrients, alone or in combination, results in improved outcomes.

Before embarking on large clinical intervention studies, it is often best to first exhaust the use of cell culture and animal models. This has been exemplified by the recent early termination of SELECT, a large clinical intervention study using selenomethionine and all-rac-α-tocopherol acetate alone and in combination for prostate cancer risk reduction, because of the apparent lack of efficacy [4]. Selenomethionine and α-tocopherol have been shown to be only marginally effective in prostate cancer animal models [5]–[9].

An additional consideration in developing natural supplements for cancer prevention or therapy is the selection of the chemical form of the test compounds. Selenomethionine is less effective than methylselenocysteine at decreasing cancer incidence and/or progression in breast [10], [11], colon [10], [12], and prostate cancer models [5], [7], [8], [13]. For vitamin E, epidemiological [14]–[17], *in vitro* [18]–[20], and animal studies [5], [6], [21]–[24] are more supportive of γ-tocopherol than α-tocopherol for decreasing prostate cancer risk, cell proliferation, or tumor development or growth. Likewise, we reported that lycopene alone is not as effective as 10% tomato powder at decreasing the development or progression of prostate cancer in two animal models [25], [26]. Although these compounds have not been particularly effective when supplemented alone, compelling evidence supports their use in combination [21], [27]–[31].

On the basis of this notion of combinatorial approaches to reduce tumor growth, we tested the ability of lycopene, methylselenocysteine, and γ-tocopherol alone and in combination to decrease the growth of androgen-dependent Dunning R-3327H rat prostate adenocarcinomas. The Dunning R-3327H model is a transplantable tumor model that originated from a spontaneous tumor in a Copenhagen rat [32]. It is an appropriate model for this study because it is a slow-growing, nonmetastatic, androgen-responsive [32], [33] tumor that responds to dietary interventions [26], [34]. We hypothesized that combinations of these micronutrients would additively or synergistically decrease tumor area and weight.

## Results

**Figure 1:**
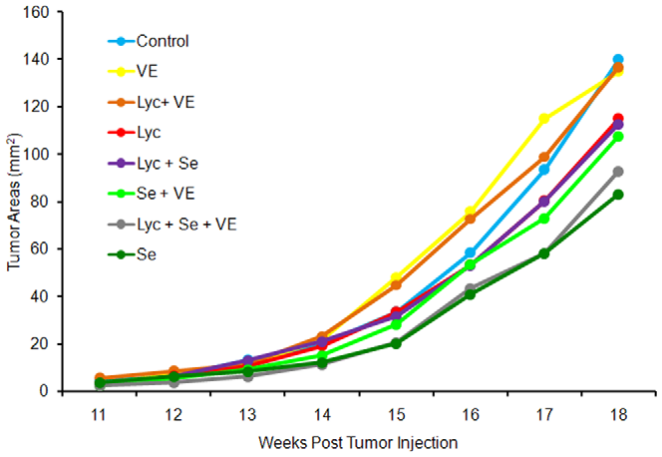
Dunning R3327-H tumor area (n = 26 to 38). Selenium consumption led to a significant decrease in tumor area (*P* = 0.003), but vitamin E and lycopene consumption did not alter tumor areas.

The following abbreviations are used to represent the diet groups: lycopene, Lyc; selenium, Se; Vitamin E, VE; Lyc + Se; Lyc + VE; Lyc + Se + VE. Tumor incidence was greater than 99%, with only one rat injection site not developing a tumor (Lyc + Se). This site was not included in the tumor area or tumor weight calculations. Selenium consumption led to a significant decrease in final tumor area (*P* = 0.003, Figure 1) and tumor weight (*P* = 0.003, Table 1). Neither lycopene nor vitamin E consumption altered final tumor area or tumor weight, and no nutrient combination interactions significantly influenced tumor growth.

**Table 1:** Final body weight, food intake, gain/food intake ratio, tumor weight, and tumor weight/final body weight ratio^1^.

Group	Final Body Weight (g)	Food Intake (g/d)	Gain/Food intake Ratio (g gained/g total food intake) ×1000	Tumor Weight (g)	Tumor Weight/Final Body Weight Ratio ×10000
Control	360±7 (19)	16.7±0.3 (19)	70.7±2.2 (19)	1.26±0.16 (38)	34.0±4.3 (38)
VE^2^	365±9 (14)	16.6±0.4 (15)	70.8±2.3 (14)	1.43±0.19 (27)	38.4±5.2 (27)
Lyc + VE	364±5 (14)	16.6±0.4 (14)	72.1±2.3 (14)	1.52±0.26 (28)	40.0±6.8 (28)
Lyc	364±11 (14)	16.7±0.3 (14)	72.4±3.6 (14)	1.13±0.16 (30)	31.2±4.6 (30)
Lyc + Se	343±8 (14)	16.2±0.2 (14)	68.8±2.9 (14)	0.96±0.14 (27)	26.8±3.9 (27)
Se + VE	359±10 (14)	16.6±0.3 (14)	68.9±2.4 (14)	1.21±0.20 (27)	32.8±5.3 (27)
Lyc + Se + VE	338±7 (14)	16.0±0.3 (15)	64.6±1.8 (15)	0.80±0.10 (29)	22.4±2.7 (29)
Se	347±9 (14)	16.1±0.4 (14)	67±2.7 (14)	0.80±0.11 (26)	21.9±3.1 (26)

1 Data are means ± SEM, ( ) sample size, selenium consumption resulted in a significant decrease in final body weight (*P* = 0.007), food intake (*P* = 0.012), gain/food intake ratio (*P* = 0.022), tumor weight (*P* = 0.003), and tumor weight/body weight ratio (*P* = 0.003).

2 Lyc, lycopene; Se, selenium; VE, vitamin E.

Diets were well accepted and consumed; nevertheless, selenium consumption led to a significant decrease in final body weight (*P* = 0.007, Table 1). Food intake varied by less than 1 g/day between diet groups (Table 1), but selenium consumption was associated with a small, yet significant, decrease in daily food intake (*P* = 0.012) and gain/food intake ratio ([body weight gained/total food intake] ×1000, *P* = 0.022, Table 1). To normalize tumor weight for body weight, we calculated the tumor weight/body weight ratio. This measure had the same *P*-value (*P* = 0.003) as the *P*-value for tumor weight alone. The correlations between body weight and tumor weight (r = 0.12) and gain/food intake ratio and tumor weight (r = 0.09) were not significant.

We used high performance liquid chromatography (HPLC) to measure hepatic lycopene, α-tocopherol, and γ-tocopherol concentrations and neutron activation analysis to quantify selenium concentrations to verify that supplementation was effective and whether nutrient accumulation interactions had occurred. All diets contained 66 mg/kg diet α-tocopherol, γ-tocopherol-supplemented diets contained 195 mg/kg diet γ-tocopherol, and lycopene-supplemented diets contained 291 mg/kg diet lycopene. Hepatic lycopene concentrations in the Lyc + VE group were increased compared with Lyc alone (Table 2, *P*<0.05). There was no difference in hepatic γ-tocopherol concentrations between vitamin E-supplemented groups or in α-tocopherol among all dietary groups. Lycopene and γ-tocopherol were not detected in nonsupplemented groups (Table 2). Neutron activation analysis showed that selenium-supplemented diets contained 1.03 mg/kg diet, whereas nonsupplemented diets contained 0.09 mg/kg diet selenium. Selenium supplementation increased hepatic selenium concentrations (Table 2, *P*<0.05), but there were no differences between selenium-supplemented groups.

**Table 2:** Hepatic lycopene, γ-tocopherol, α-tocopherol, and selenium levels^1^.

Group	Total^2^ Lycopene (µg/g)	γ-tocopherol^3^ (µg/g)	α-tocopherol (µg/g)	Selenium (µg/g)
Control	ND^4^	ND	76±4	0.83±0.03
VE^5^	ND	5.5±0.6	70±4	0.85±0.02
Lyc + VE	203±13*	6.1±1.1	77±7	0.79±0.06
Lyc	148±20	ND	72±3	0.77±0.06
Lyc + Se	171±9	ND	78±7	1.15±0.06**
Se + VE	ND	6.4±2.0	64±5	1.16±0.02**
Lyc + Se + VE	149±13	3.9±0.7	72±3	1.12±0.04**
Se	ND	ND	67±5	1.17±0.03**

1 Data are means ± SEM (n = 6),

* *P*<0.05 vs. Lyc group,

** *P*<0.05 vs. control group.

2 All-*trans* + *cis* isomers.

3 Limit of detection 0.6 µg/g.

4 Not detectable.

5 Lyc, lycopene; Se, selenium; VE, vitamin E.

Because of the importance of androgens in stimulating the growth of Dunning R3327-H tumors, we evaluated whether selenium consumption altered serum testosterone or dihydrotestosterone concentrations. There were no differences in either serum androgen among dietary groups (Table 3). Other potential mechanisms for decreased tumor growth with selenium consumption include altered tumor proliferation and/or apoptotic rates. We used immunohistochemistry to calculate a tumor proliferation index and count tumor apoptotic cells; however, there were no significant differences in these measures among dietary groups (Table 3). We next measured the activity of the selenoenzyme, glutathione peroxidase, to determine whether selenium consumption induced this enzyme in the prostate and/or tumor. We found no difference in glutathione peroxidase activity in the prostate or tumor with selenium consumption compared with animals fed the control diet (data not shown).

**Table 3:** Serum testosterone, dihydrotestosterone levels, tumor proliferation index, and apoptotic cell numbers^1^.

Diet Group	Serum Testosterone (ng/mL)	Serum Dihydrotestosterone (pg/mL)	Tumor Proliferation Index (%)	Tumor Apoptotic Cell Number
Control	1.4±0.2 (17)	88±9 (14)	45.2±4.7 (10)	8.2±0.9 (20)
VE^2^	1.2±0.1 (13)	81±8 (13)	44.2±4.8 (10)	9.3±1.1 (20)
Lyc + VE	1.1±0.1 (14)	83±8 (14)	44.9±3.1 (10)	7.5±0.9 (20)
Lyc	1.2±0.1 (11)	81±10 (12)	43.7±5.5 (10)	9.6±0.9 (20)
Lyc + Se	1.4±0.1 (10)	86±6 (12)	50.1±4.5 (10)	7.9±0.6 (20)
Se + VE	1.1 ±0.1 (14)	80±8 (14)	47.6±5.0 (10)	10.4±1.0 (20)
Lyc + Se + VE	1.3±0.1 (13)	81±7 (15)	54.2±3.7 (10)	7.3±0.7 (20)
Se	1.1±0.1 (12)	75±8 (13)	46.7±4.4 (10)	7.6±0.6 (20)

1 Data are means ± SEM, ( ) sample size, no significant differences for any of these parameters.

2 Lyc, lycopene; Se, selenium; VE, vitamin E.

Selenium treatment, or consumption, has been reported to alter androgen receptor signaling or expression [35]–[45]. Thus, we measured androgen receptor and probasin mRNA expression in the normal prostate and tumor of all dietary groups. Probasin (prostate basic protein) is a prostate-specific, androgen-response gene [46]. There was no difference in androgen receptor or probasin mRNA expression between diet groups in either tissue (data not shown).

We next examined whether selenium consumption altered the mRNA expression of three selenium-associated proteins: SepP, Sep15, and SBP2. We selected these selenium-associated proteins for two reasons. First, their expression has been reported to decrease in prostate cancer tissue and/or cell lines compared with normal tissue or less-advanced prostate cancer cell lines [47]–[49]. In addition, their expression is altered by androgens [47], [50]–[54]. Nonetheless, there was no difference in SepP, Sep15, and SBP2 mRNA expression between diet groups in either the prostate or the tumor (data not shown).

Because we did not find differences in SepP, Sep15, and SBP2 mRNA expression with selenium consumption, we measured mRNA expression in prostates and tumors from finasteride-treated and castrated rats from a previous study [26]. This was done to ensure that the expression of SepP, Sep15, and SBP2 was altered by androgens as reported previously [47], [50], [52]–[54]. Finasteride is a 5α-reductase inhibitor that prevents the conversion of testosterone to dihydrotestosterone, making it a weak androgen ablation therapy. In the prostate, castration decreased prostate Sep15 levels (*P* = 0.0003, Table 4), whereas finasteride treatment decreased prostate SepP expression (*P* = 0.008, Table 4). In the prostate, SBP2 expression was increased by both finasteride treatment (*P* = 0.002, Table 4) and castration (*P* = 0.008, Table 4). In the tumor, castration decreased Sep15 expression levels (*P* = 0.003, Table 4), and there were no alterations in SBP or SepP expression levels or effects of finasteride treatment.

**Table 4:** Prostate and tumor selenoprotein 15 (Sep15), selenoprotein P (SepP), and selenium binding protein 2 (SBP2) mRNA expression in castrated and finasteride treated rats^1^.

	Prostate			Tumor		
Group	Sep15 (n = 5–6)	SepP (n = 5–6)	SBP2 (n = 5–6)	Sep15 (n = 11–13)	SepP (n = 11–13)	SBP2 (n = 10–13)
Control (Cas)	1.0±0.1	1.0±0.2	1.0±0.1	1.0±0.1	1.0±0.1	1.0±0.1
Castrated	0.4±0.1**	0.9±0.1	2.0±0.1**	0.7±0.1**	1.1±0.1	1.1±0.1
Control (Fin)	1.0±0.2	1.0±0.1	1.0±0.1	1.0±0.1	1.0±0.1	1.0±0.1
Finasteride	0.8±0.1	0.6±0.1*	1.8±0.2*	1.0±0.1	1.2±0.1	0.8±0.1

1 Values are means ± SEM, asterisks indicate significant differences compared with the corresponding control,

* *P*<0.05,

** *P*<0.01.

## Discussion

The major finding of this study was that dietary methylselenocysteine, but not lycopene or γ-tocopherol, reduced prostate Dunning R3327-H adenocarcinoma growth in male Copenhagen rats. Contrary to our hypothesis and others' findings [21], [27]–[31], we did not observe additive or synergistic reductions in tumor growth with combinations of the different micronutrients. The ineffectiveness of lycopene alone is, in general, consistent with our [25], [26] and others' findings [21], [22], [55] in rodent prostate cancer models. Unlike the inhibition seen previously in transgenic prostate cancer models [23], [24], γ-tocopherol did not inhibit tumor growth in the present study. These differences may indicate that γ-tocopherol prevents development of prostate cancer, which cannot be investigated in this model, rather than inhibits growth of prostate tumors.

The dietary level of lycopene is the same as our previous rat prostate cancer studies [25], [26], and was originally selected to yield lycopene tissue levels similar to what occurs in humans [56], [57]. We originally set out to supplement the diets with 2 mg Se/kg diet because in breast cancer models this level was able to decrease cancer incidence by 50% and did not result in adverse outcomes, such as decreased food intake or body weights [10]. However, the diet analysis revealed that the diet contained 1 mg Se/kg diet. For γ-tocopherol, we chose 200 mg γ-tocopherol/kg diet based on the finding that 150 mg γ-tocopherol/kg diet increased both α-tocopherol and γ-tocopherol tissue levels in vitamin E deficient rats [58].

The small but significant decreases in body weight, food intake, and gain/food intake ratio with methylselenocysteine consumption was unexpected. Of 20 publications identified [8], [12], [13], [59]–[75], only one [13], which fed 1 to 3 mg Se/kg diet as methylselenocysteine, or vegetable powder that accumulates this compound, for ≥10 weeks, reported a significant decrease in body weight. However, the significance of selenium consumption's reduction in tumor weight was not altered when we controlled for body weight by calculating the tumor/body weight ratio. Further support is provided by correlations between final body weight and tumor weight or gain/food intake ratio and tumor weight that were not statistically significant. Overall, we cannot rule out that decreased body weight, food intake, gain/food intake ratio, or cytoxic effects of selenium may have played a small role in the decrease in tumor growth.

For lycopene, selenium, and vitamin E status, we measured hepatic concentrations, which are a good indicator of prostate and tumor lycopene [26] and selenium concentrations [8] as well as overall body status. Selenium consumption resulted in a 42% increase in hepatic selenium concentrations, similar to what has been reported previously [8]. There was no increase in hepatic α-tocopherol concentrations with γ-tocopherol supplementation as reported previously [58]. Similar to our results, a recent publication also found no alteration in plasma α-tocopherol concentrations in transgenic male rats fed γ-tocopherol [24]. We found a significant 37% increase in hepatic lycopene concentrations with γ-tocopherol consumption that we believe may be due to the antioxidant action of γ-tocopherol, which could prevent oxidation of lycopene. However, hepatic lycopene concentrations were not increased in the Lyc + Se + VE group compared with the Lyc group. More research on combinations of these nutrients is needed to characterize these interactions.

Despite the reduction in tumor weight and final tumor area with selenium consumption, tumor proliferation or apoptosis rates were unaltered. The slopes of the increase in tumor area from week 17 to week 18 (Figure 1) were similar among all diet groups, indicating similar growth rates at the end of the study. Thus, it is possible that if we had terminated the study earlier, when growth rates were different, we may have found differences in tumor proliferation and apoptosis rates. Selenium consumption also did not alter glutathione peroxidase activity in the prostate or tumor, similar to previous findings in the liver and colon of mice [76]. To the best of our knowledge, we are the first to determine that selenium consumption does not alter glutathione peroxidase activity in the prostate or prostate tumors.Selenium consumption did not alter androgen receptor, probasin, SBP2, Sep15, or SepP prostate or tumor mRNA expression. Our finding of no alteration of androgen receptor expression with methylselenocysteine consumption differs from consumption and intraperitoneal injection of methylselenocysteine that decreased androgen receptor expression in the rat prostate [45] and LNCaP tumor xenographs in nude mice [44], respectively. The reason for the difference in results is not clear but could be related to our lower selenium dose, animal model, and/or method of gene expression measurement. To the best of our knowledge, we are the first to determine whether selenium consumption alters expression of SBP2, Sep15, and SepP in the prostate or prostate tumors. There are two very similar SBPs in mice, SBP1 and SBP2 [77], but in both rats and humans, only one SBP has been identified. The human gene is SBP1 [78], and the rat gene is SBP2 [79]. Our finding of no differences in SBP2 with selenium is in agreement with the lack of an effect of selenium consumption on mouse hepatic SBP1 protein levels [80]. However, it differs from the increased SBP1 expression with methylselenocysteine treatment of normal human ovarian surface cells *in vitro* and decreased expression in three of the four human ovarian cancer cell lines [51]. Our finding that Sep15 expression was not altered by selenium consumption or deficiency is consistent with previous studies in which mouse hepatic Sep15 protein [76], [81] and mRNA levels [81], [82] were not altered with selenium consumption or deficiency. We did not find an alteration in SepP in either tissue, and hepatic SepP expression levels previously were reported to be decreased in selenium-deficient rats [83], but not in the colon of selenium-deficient mice[82].

In castrated or finasteride-treated rats, prostate SBP2 mRNA expression was increased. This is in agreement with work investigating the effects of dihydrotestorone treatments of prostate cancer cells in culture [47], [50]. In contrast, neither castration nor finasteride treatment altered tumor SBP2 expression. Castration significantly decreased prostate and tumor Sep15 expression similar to the decrease in rat ventral prostate Sep15 expression following castration reported previously [52]. It should be noted that this was the only gene altered by castration in both the prostate and tumor. Finasteride treatment decreased prostate SepP expression. Previously, castration was reported to decrease kidney but not hepatic SepP expression in mice [53]. In addition, in LNCaP cells, *in vitro* synthetic androgen R1881treatment increased SepP expression [54]. Thus, it appears that SepP expression is stimulated by androgens. Overall, Dunning tumor gene expression seemed to be less responsive to androgen ablation than prostate gene expression. This may be due to the location of the tumor or its altered physiology.

In conclusion, only methylselenocysteine consumption decreased tumor weight and final tumor area, and there was no additional benefit of consuming lycopene and/or γ-tocopherol. Methylselenocysteine consumption did not alter serum androgens; tumor proliferation rates; tumor apoptosis rates; glutathione peroxidase activity; or androgen receptor, probasin, Sep15, SepP, or SBP2 expression. Thus, another mechanism of action for selenium's ability to decrease tumor growth is yet to be elucidated. Castration decreased prostate and tumor Sep15 expression and increased prostate SBP2 expression. Finasteride treatment decreased prostate SepP expression and increased prostate SBP2 expression.

The SELECT results show the importance of conducting preclinical animal models and publishing those results even if nonsignificant effects are seen [84], [85]. Evidence in preclinical models suggests that all forms of selenium (selenomethionine, selenite, etc.) should not be lumped together because of their varied efficacy. Instead, studies should be interpreted according to what selenium compound was administered with the current study adds to the methylselenocysteine and prostate cancer literature [86]. Our lycopene and/or γ-tocopherol results add to growing evidence suggesting that isolated nutrients, alone or in combination, may not be an effective strategy for decreasing tumor growth [85], [87], [88].

## Materials and Methods

### Ethics Statement

The University of Illinois Laboratory Animal Care Advisory Committee approved all animal procedures.

### Experimental Diets

Study rats were fed the control diet for 1 week after receipt and then were randomized into one of eight AIN-93G [89] based powder study diet groups (Table 5) for 4 to 6 weeks prior to tumor implantation. Diets were balanced for protein, fat, energy, and fiber and stored at 4°C in the dark. All diets were provided *ad libitum*. Diets were replaced and food intakes calculated every 48 h. Fresh diets were prepared monthly (all diet ingredients from Harlan Teklad, Madison, WI, unless otherwise noted).

**Table 5:** AIN-93G-based lycopene, selenium, and vitamin E diet formulations.

Diets Composition (g)	Control	Lyc^1^	Se	VE	Lyc + Se	Lyc + VE	Se + VE	Lyc + Se + VE
Cornstarch	395	395	385	395	385	395	385	385
Casein	200	200	200	200	200	200	200	200
Maltodextrin	132	132	132	132	132	132	132	132
Sucrose	100	100	100	100	100	100	100	100
Soybean Oil^2^	70	70	70	70	70	70	70	70
Cellulose	50	50	50	50	50	50	50	50
Mineral Mix	35	35	35	35	35	35	35	35
Vitamin Mix^3^	10	10	10	10	10	10	10	10
L-Cystine	3	3	3	3	3	3	3	3
Choline Bitartrate	2.5	2.5	2.5	2.5	2.5	2.5	2.5	2.5
Lyc Beadlets^4^	-	2.5	-	-	2.5	2.5	-	2.5
Placebo Beadlets	2.5	-	2.5	2.5	-	-	2.5	-
γ-Tocopherol	-	-	-	0.2	-	0.2	0.2	0.2
Se Premix^5^	-	-	10	-	10	-	10	10
Total	1000	1000	1000	1000	1000	1000	1000	1000

1 Lyc, lycopene; Se, selenium; VE, vitamin E.

2 Tocopherol-stripped.

3 27 mg/kg diet as all-rac-α-tocopherol acetate.

4 10% lycopene.

5 200 mg/kg of selenium in cornstarch as methylselenocysteine.

Tocopherol-stripped soybean oil (Dyets, Inc., Bethlehem, PA) was used to limit exogenous, nonsupplemented tocopherol levels. In addition, the vitamin mix (Harlan Teklad TD.05521) was formulated to provide the National Research Council requirement for rats (27 mg/kg diet as all-rac-α-tocopherol acetate) [90] instead of the 75 mg/kg diet normally provided by AIN93-G diets [89]. Vitamin E was supplemented as γ-tocopherol (D-Gamma Tocopherol 90, Eisai Food & Chemical Co., Ltd., Tokyo, Japan) at 200 mg/kg diet in VE groups. Lycopene was added to diets in the form of 10% lycopene beadlets (redivivo^TM^, DSM, Basel, Switzerland) to deliver 250 mg/kg diet as we have done previously [25], [26]. Equivalent amounts of placebo beadlets (DSM) were used in diets not containing lycopene. Selenium was supplemented in the form of methylselenocysteine (Se-Methylseleno-L-cysteine, PharmaSe, Lubbock, TX) at 1 mg/kg diet of elemental selenium.

### Rats

One hundred and twenty-six ≈100 gram male Copenhagen rats (Cop 2331; Harlan, Indianapolis, IN) were individually housed in wire-bottomed cages. Rats were monitored daily and weighed twice weekly. Two control rats were euthanized prior to study completion because of high tumor burden. Five other rats were euthanized prior to study completion for health reasons unrelated to tumor growth (1 Se, 1 VE, 1 Lyc + Se, 1 Lyc + VE, 1 VE + Se); 119 rats completed the study. The study was carried out in three cohorts (n = 6 or 7) with 6 or 7 control rats per cohort. Cohorts were necessary because the rats could not be purchased at one time. Cohort was used as a covariate in statistical analysis to control for possible differences between cohorts.

### Dunning R3327-H Tumor Implantations

Ten ≈150 g male Copenhagen donor rats (Harlan, Indianapolis, IN) had tumor pieces coated in Matrigel™ Basement Membrane Matrix (BD Biosciences, Bedford, MA) subcutaneously implanted in their hind flanks. Tumors were allowed to grow for 15 to 20 weeks and then were harvested for implantation into study rats.

Tumors were sterilely harvested from donor rats by removing the tumor capsule, necrotic areas, and large blood vessels. The remaining tissue was mixed with Hanks Balanced Salt Solution, minced, pushed through a 20 mesh sieve, and placed in a 50 mL centrifuge tube. The solution was centrifuged at 200×*g* for 10 minutes, and the supernatant was removed. The remaining tumor pellet was mixed with Matrigel™ at a concentration of 100 mg tumor/mL, and 0.2 mL (≈20 mg of tumor) was injected with 19-gauge needles into both rear flanks of study rats. Tumors were allowed to grow for ≈18 weeks until study termination. Tumors were palpated weekly, and tumor length and width were measured with calipers. From these measurements, tumor area was calculated using the formula for area of an ellipse: area = π*(length/2)*(width/2). When a tumor was not found, a zero was recorded for that week. At study termination, rats were anesthetized with CO_2_, blood was taken via cardiac puncture, tumors were removed and cleaned, and tumor weights were recorded. Tumor and selected tissues were flash frozen in liquid nitrogen and then stored in a −80°C freezer. In addition, a piece of tumor was placed in 10% formalin (Sigma-Aldrich) for 24 to 48 hours before being moved to 70% ethanol.

### Hepatic and Diet Lycopene and Vitamin E Quantification

For liver, 0.1 g of tissue was placed into a 50-mL glass centrifuge tube. Ethanol containing 0.1% butylated hydroxytoluene (3.5 mL) was added along with deionized distilled water (1.5 mL) and ascorbic acid (0.25 g). Tocol (Matreya, Pleasant Gap, PA) and β-carotene (Sigma-Aldrich, St. Louis, MO) were added as internal standards for tocopherols and carotenoids, respectively. Saturated potassium hydroxide (1 mL) was then added, and samples were vortexed and saponified at 60°C for 30 min in a water bath. Ascorbic acid was necessary to prevent oxidation of vitamin E during saponification as described previously [91]. Test tubes were then removed from the water bath and placed on ice, and deionized distilled water (2 mL) was added prior to extraction three times with hexane (6 mL). The extracts were dried down in a Speedvac concentrator (model AES1010; Savant, Farmingdale, NY), covered with argon, and stored at −20°C for less than 48 hours before HPLC analysis. The procedure was the same for diet extraction except 0.25 g of diet was extracted, and internal standards were not used. Samples were analyzed by using an isocratic HPLC method described previously with minor modifications [92]. The mobile phase consisted of acetonitrile/dioxane/2-propanol/methanol/triethylamine (800/150/25/25/1) with 200 mM ammonium acetate in the alcohol component. Samples were reconstituted in ethyl acetate: mobile phase (100∶35). The HPLC system consisted of a Varian Prostar 325 UV-Vis Dual Wavelength Detector (Walnut Creek, CA) monitoring 300 nm and 450 nm wavelengths, a Varian 410 Autosampler, Rainin SD-200 Dynamax pumps, a precolumn (Upchurch Scientific, Oak Harbor, WA) packed with ODS C-18 (Alltech Associates, Deerfield, IL), a C-18 Waters Spherisorb 3 µM ODS2 4.6×150 mm column (Milford, MA), and a CERA 250 column cooler (Baldwin Park, CA) set at 16°C for the 25-minute run duration. Our laboratory routinely participates in the National Institutes of Standards in Technology micronutrient proficiency testing program, and our values for lycopene, α-tocopherol, and γ-tocopherol normally are within one standard deviation of the median.

### Selenium and Serum Androgen Quantification

Hepatic and diet selenium concentrations were determined by neutron activation analysis [93] at the University of Missouri Research Reactor Center (reported as wet weight concentrations). Serum testosterone and dihydrotestosterone concentrations were determined with DSL-4000 and DSL-9600 radioimmunoassay kits, respectively (Diagnostic Systems Laboratories Inc., Webster, TX) according to the manufacturer's instructions.

### Tumor Histology and Immunohistochemistry

At study termination**,** a piece of Dunning R3327-H tumor was fixed in 10% formalin (Sigma-Aldrich) for 24 to 48 hours before being moved to 70% ethanol. Samples were then processed overnight in a vacuum infiltration processor E300, (Sakura Finetek, Torrance, CA), paraffin embedded by using a Tissue Tek TEC Embedding Center (Sakura Finetek), sectioned at 3 µM by using a HM315 Microm Microtome, and transferred to positively charged barrier slides (BioGenex, San Ramon, CA). Slides were deparafinized and hydrated by using a Sakura DRS 2000 followed by methanol 3% H_2_O_2_ solution for 15 min. To unmask proliferating cell nuclear antigen (PCNA), slides were placed in citrate buffer (pH 6) in a decloaking chamber for 30 seconds at 125°C and 10 seconds at 90°C. The slides were then transferred to a Biogenex i6000 immunostainer, where the slides were blocked for 10 minutes with Power Block™ (BioGenex) then blocked with avidin and biotin for 15 minutes each. Rabbit anti-PCNA antibody (Dako, Carpinteria, CA) was incubated for 30 minutes followed by the DakoCytomation LSAB2 System. The LSAB2 Biotinylated Link was incubated for 15 min, the LSAB2 Streptavidin-HRP label for 15 minutes, followed by 3,3′-diaminobenzidine tetrahydrochloride (BioGenex) for 5 minutes. The slides were then counterstained with Mayer's hematoxylin (BioGenex) for 1 minute. The PCNA images were blindly captured and quantified as described previously [26]. Two representative images per tumor were captured, and a proliferative index percentage, (PCNA positive/total nuclei counter) ×100, was calculated. The proliferative index reported is the mean of two proliferative index percentages per tumor.

Apoptosis was measured with an Apoptag® Peroxidase In Situ Apoptosis Detection Kit S7100 (Chemicon International, Temecula, CA) according to the manufacturer's instructions except that slides were counterstained with Mayer's hematoxylin rather than methyl green. Images were blindly captured and quantified as described previously [26]. Two representative images, without artifacts, from each tumor were quantified by counting the number of nuclei at 400× magnification. The apoptotic cell number reported is the mean of the two apoptotic nuclei values per tumor.

### Real-Time PCR

Castration and finasteride tissues were from two separate studies described previously [26]; therefore, controls were needed as a comparison to both groups. Tumors and dorsolateral plus anterior prostate lobes were extracted with the PureLink Micro-to-Midi Total RNA Purification System (Invitrogen, Carlsbad, CA) according to the manufacturer's instructions. Tumors and dorsolateral prostates from the Lyc, Se, and VE groups were extracted using Trizol® reagent (Invitrogen, Carlsbad, CA) according to the manufacturer's instructions. After extraction, the sample was treated with DNase (DNase I, New England Biolabs, Ipswich, MA) following the manufacturer's typical reaction instructions except 20 µg of RNA and four units of DNAse I were used. The RNA was then ethanol precipitated [94] and resuspended in RNase-free water. The RNA was run on an agarose gel to examine RNA integrity, and samples of sufficient quality were used to synthesize cDNA with High-Capacity cDNA Reverse Transcription Kits (Applied Biosystems, Foster City, CA) and an Eppendorf Mastercycler Gradient (Westbury, NY).

Primers were selected across exon boundaries by using Primer Express software. Primers (MWG, Huntsville, AL) were validated to ensure efficiency and amplification of a single amplicon. The primers selected were: androgen receptor forward AGAACTACTCCGGACCTTATGGG, reverse TGGTCCCTGGTACTGTCCAAAC; probasin forward AGAAATGACGTTACACGAGTGGC, reverse CAGTTGCCTGCCTTTCGC; SepP forward AGCCAGCTGATACCTGTGCC, reverse CGCAGGTCTTCCAATCTGGA; Sep15 forward AACCCAAACTGTTCAGAGGTCTACA, reverse GGTCTGAGCCTCGAACATACTTG; and SBP2 forward GCCTCACTGGGCAGATCTTC, reverse CAGAGCCTCCTTTAACAATGCTG.

The rRNA L7a was used as an internal control by using previously published sequences [95]. We verified equivalent expression between diet and treatment groups in both tissues examined. Real-time PCR was performed with SYBR Green Mastermix (Applied Biosystems), a 7900 Fast HT Real-Time PCR machine (Applied Biosystems), and 4 ng prostate or tumor cDNA template according to the manufacturer's instructions. The relative mRNA abundance was determined by using the comparative critical threshold method and is expressed relative to the control diet group [96].

### Gluathione Peroxidase Activity

Frozen dorsolateral prostate and tumor samples were resuspended in cold sodium phosphate buffer (0.1M, pH 7.0) and lysed by sonication. Total glutathione peroxidase activity was determined using a standard coupled spectrophometric method based on the consumption of reduced NADPH measured spectrophotometrically by absorbance at 339 nm in a reaction containing reduced glutathione, glutathione reductase, tissue lysates, and hydrogen peroxide as described elsewhere [97].

### Statistics

Tumor weight; final tumor area; final body weight; daily food intake; gain/food intake ratio; tumor weigh/body weight ratio; proliferation indexes; apoptotic cell numbers; and Lyc, Se, and VE tumor and prostate gene expression were analyzed by using dummy-coded multiple linear regression to look for the effects of lycopene, selenium, and vitamin E consumption and interactions among these nutrients [98], [99]. Cohort was used as a covariate (effects coded) but was retained only in the models for final tumor area, daily food intake, and gain/food intake ratio. Hierarchical analysis revealed that covariate interaction terms and treatment interaction terms did not account for a significant amount of variance; thus, these terms were removed from all the models. For tumor weight; final body weight; tumor weight/body weight ratio; proliferation indexes; apoptotic cell numbers; and Lyc, Se, and VE gene expression, the final models contained only the three main treatment variables (Lyc, Se, and VE). The natural logs of both tumor area and tumor weight were used to correct violations of model assumptions. Tumor weight analysis with proc univariate in SAS identified five very large, outlying tumors (greater than 1.5× interquartile range). These five tumors (2 Se, 1 VE, 1 Se + VE, 1 Lyc + Se + VE) were excluded from tumor weight and final tumor area analysis. One rat (Lyc) was excluded from gain/food intake ratio analysis because of weight loss at the end of the study. Serum testosterone, serum dihydrotestosterone, hepatic lycopene, hepatic vitamin E, and hepatic selenium concentrations were analyzed using ANOVA with Dunnett's test. Correlations of final body weight with tumor weight and final body weight with gain/food intake ratio were performed using the proc corr command in SAS. Castration and finasteride treatment expression levels were analyzed using two-sample t-tests. Natural logs of a number of castration and finasteride treatment gene expression levels were used to correct for assumption violations. When this transformation did not correct assumption violations, the Wilcoxon Rank Exact Sum test [100] was used. All statistics were performed using SAS 9.1 (Carey, NC), and *P*<0.05 was considered significant.
